# Preparation, Characterization, and Biological Evaluation of Poly(Glutamic Acid)-*b*-Polyphenylalanine Polymersomes

**DOI:** 10.3390/polym8060212

**Published:** 2016-06-02

**Authors:** Evgenia Vlakh, Anastasiia Ananyan, Natalia Zashikhina, Anastasiia Hubina, Aleksander Pogodaev, Mariia Volokitina, Vladimir Sharoyko, Tatiana Tennikova

**Affiliations:** 1 Institute of Chemistry, Saint-Petersburg State University, Universitesky pr. 26, 198504 St. Petersburg, Russia; vlakh@mail.ru (E.V.); zurako@ukr.net (A.H.); pogodaev@gmail.com (A.P.); volokitinamariya@yandex.ru (M.V.); sharoyko@gmail.com (V.S.); 2 Institute of Macromolecular Compounds, Russian Academy of Sciences, Bolshoy pr. 31, 199004 St. Petersburg, Russia; ananas27@rambler.ru (A.A.); nzashihina@bk.ru (N.Z.)

**Keywords:** poly(amino acid)s, amphiphilic copolymers, polymer particles, polymersomes, encapsulation, biodegradation, cell uptake

## Abstract

Different types of amphiphilic macromolecular structures have been developed within recent decades to prepare the polymer particles considered as drug delivery systems. In the present research the series of amphiphilic block-copolymers containing poly(glutamatic acid) as hydrophilic, and polyphenylalanine as hydrophobic blocks was synthesized and characterized. Molecular weights for homo- and copolymers were determined by gel-permeation chromatography (GPC) and amino acid analysis, respectively. The copolymers obtained were applied for preparation of polymer particles. The specific morphology of prepared polymerosomes was proved using transmission electron microscopy (TEM). The influence on particle size of polymer concentration and pH used for self-assembly, as well as on the length of hydrophobic and hydrophilic blocks of applied copolymers, was studied by dynamic light scattering (DLS). Depending on different experimental conditions, the formation of nanoparticles with sizes from 60 to 350 nm was observed. The surface of polymersomes was modified with model protein (enzyme). No loss in biocatalytic activity was detected. Additionally, the process of encapsulation of model dyes was developed and the possibility of intracellular delivery of the dye-loaded nanoparticles was proved. Thus, the nanoparticles discussed can be considered for the creation of modern drug delivery systems.

## 1. Introduction

The development of modern drug delivery systems to solve the problem of directed transport at cellular and sub-cellular scales reduces both the probability of loaded drug degradation and its high toxicity in the body [1]. The variety of polymer materials opens up wide possibilities to create such systems of tunable morphology appropriate to the size of the organism. Different types of carriers have been constructed for drug delivery applications, including polymer conjugates, nanogels, along with nano- and microparticles of different morphologies [2,3,4]. Recently, elevated attention has been paid to the polymer nanovesicles of a core-shell structure with double layer liposome-like membranes [5,6,7,8]. The structure of such a polymer shell has much in common with that of a cell membrane, which can enhance the cell permeability for developed nanoparticles. These nanocarriers, composed of the amphiphilic block-copolymers, are known under the name polymersomes and, compared to liposomes, demonstrate higher membrane stability (mainly due to the membrane thickness, which can exceed 9 nm) and tunable membrane properties [9,10,11]. The thicker membrane prolongs the stability of the nanocarrier in a blood flow and enhances the therapeutic efficacy of the loaded drug [11]. Furthermore, the amphiphilic membrane of polymersomes provides the advantage to integrate both hydrophilic and hydrophobic compounds, such as drugs, genes, imaging agents, *etc*. [1,4,7]. The unique controllable structure of polymersomes allows the construction of nanovesicles with stimuli response depending on temperature, pH value, or ionic strength change, which can disassemble and release the encapsulated drug [12,13]. 

Different types of amphiphilic structures have been developed in recent decades, including dendritic compounds, graft, di-, and triblock copolymers. PEG is widely used as the most common neutral hydrophilic constituent of di- and triblock copolymers, or as a fragment of polymer brushes due to its low toxicity and high stability [14,15]. A lot of polymers are applied as building blocks for polymersomes, namely, polypropylene oxide, polylactic acid, polybutadiene [16], *etc*. Despite the wide diversity of building blocks for the structures discussed, the polypeptides represent one of the most attractive classes of polymers for polymersome formation. 

These polymers combine the advantages of synthetic polymers with peptides’ ability to form secondary structure, biocompatibility, functionality, and biodegradability due to their units produced as a result of degradation and representing the metabolic substances [3,17,18,19]. Moreover, it is well known that polypeptides can change their conformation under the variation of temperature or pH that can be used for construction of stimuli-responsive polymersomes [20]. The variety of functional groups located on the polymersome surface provides the possibility of nanocarrier modification with bioligands to create the systems for controlled drug delivery. 

There are two main methods for polypeptide synthesis, e.g., the step-wise in-solution or solid-phase condensation of activated amino acids, and the ring-opening polymerization (ROP) of α-amino acid *N*-carboxyanhydrides (NCA) [18,21,22]. The first way is suitable for synthesis of heteropeptides with well-defined sequences and lengths up to 100 residues. However, this approach is not appropriate for direct preparation of large polypeptides, especially homopolypeptides, because of its laborious, time-consuming, economic inefficiency. The most expedient way for synthesis of long chain homopolypeptides, random polypeptides, graft-, or block polypeptides, is the ring-opening polymerization (ROP) of *N*-carboxyanhydrides of α-amino acids. This approach represents a reliable method to obtain polypeptides of controllable molecular weight and low polydispersity index with no racemization [23]. A number of block copolypeptides, which are able to self-assemble into micelles or vesicles, was described in the literature. For example, Kim *et al.* [24] described the synthesis of poly(l-glutamic acid)-*b*-poly(l-phenylalanine) copolymers with small hydrophobic blocks consisted of 2–7 phenylalanine units for the preparation of particles. Holowka *et al.* [25] reported the development of charged polypeptide vesicles based on poly(l-lysine)_60_-*b*-poly(l-leucine)_20_, poly(l-glutamic acid)_60_-*b*-poly(l-leucine)_20_ or poly(l-argenine)_60_-*b*-poly(l-leucine)_20_ polypeptides appropriate for drug encapsulation. However, no consideration of the effect of block length or conditions of self-assembly on particle properties was given in these papers [24,25]. In Huang *et al.* [26] the influence of block copolymer length on the formation of poly(l-lysine)-*b*-poly(l-tyrosine) particles was discussed. The investigation of biocompatibility of hydrogels or particles based on block copolypeptides containing l-lysine, l-glutamic acid, or l-argenine for the building of hydrophilic blocks, and l-leucine for the construction of hydrophobic ones, revealed the absence of cytotoxicity [27,28,29]. Nevertheless, not too much attention was paid to the cytotoxicity of high molecular weight block copolypeptides, which are more prospective considering their higher stability and prolonged release in comparison to the low molecular weight analogues. 

In the current research the series of poly(l-glutamic acid)-*block*-poly(l-phenylalanine) (PGlu-*b*-PPhe) samples with polypeptide blocks containing up to 117 l-glutamic acid and 165 l-phenylalanine residues was obtained and used for the preparation of nanoparticles. The peculiarities of morphology of constructed nanoparticles were investigated. The biodegradation of PGlu-*b*-PPhe nanoparticles was evaluated using *in vitro* degradation in a model enzymatic system and human blood plasma. The cytotoxicity of polymersomes obtained was tested using two cell lines, namely, HEK and Caco-2. Additionally, the process of encapsulation of model dyes was developed and the possibility of intracellular delivery of the dye-loaded nanoparticles was proved. 

## 2. Materials and Methods 

### 2.1. Materials

γ-benzyl-l-glutamate, l-phenylalanine, triphosgene, α-pinene, n-hexylamine (HEXA), trifluoromethanesulfonic acid (TFMSA), trifluoroacetic acid (TFA), *N*-hydroxysuccinimide (NHS), 1-Ethyl-3-(3-dimethylaminopropyl)carbodiimide (CDI), bromophenol blue, rhodamine 6g, 3-(4,5-dimethylthiazol-2-yl)-2,5-diphenyltetrazolium bromide(MTT), and other reagents were purchased from Sigma-Aldrich (Darmstadt, Germany) and used as received. 1,4-dioxane, n-hexane, *N*,*N*-dimethylformamide (DMF), dimethyl sulfoxide (DMSO), ethyl acetate, methanol, and other solvents were purchased from Vecton Ltd. (St. Petersburg, Russia) and distilled before application. All salts used for buffers preparation were also purchased from Vecton Ltd. and were of ACS reagent grade. The buffer solutions were prepared by dissolving salts in distilled water and additionally purified by filtration through a 0.45-μm membrane microfilter Milex, Millipore Inc. (Hietzinger, Austria). Vivaspin concentrators (30,000) used for ultrafiltration were products of Sartorius (Goettingen, Germany). The Spectra/Pore^®^ (MWCO: 1000) dialysis bags were purchased from Spectra (Rancho Dominguez, CA, USA).

Human embryonic kidney cells (HEK 293) and human epithelial colorectal adenocarcinoma cells (Caco-2) were obtained from BioloT (St. Petersburg, Russia) and were grown in Dulbecco’s Modified Eagle’s Medium (BioloT, St. Petersburg, Russia) containing 10% (*v*/*v*) heat-inactivated fetal bovine serum (FBS, HyClone Laboratories, Logan, UT, USA), 1% l-glutamine, 1% sodium pyruvate, 50 U/mL penicillin, and 50 μg/mL streptomycin (BioloT). 

### 2.2. Instrumentation

The structure and purity level of synthesized NCAs were confirmed by ^1^H NMR. Spectra were recorded at 298 K using a Bruker 400 MHz Avance instrument (Karlsruhe, Germany) and СDCl_3_. Gel permeation chromatography (GPC) measurements were performed on Shimadzu LC-20 Prominence system with refractometric RID 10-A detector (Kyoto, Japan) using 7.8 mm × 300 mm Styragel Column, HMW6E, 15–20 µm bead size (Waters, Milford, MS, USA). The analysis was carried out at 60 °C using DMF with 0.1 M LiBr as eluent. The mobile phase flow rate was 0.3 mL/min. Molecular weights and molecular weight distributions for γ-Glu(Bzl) homopolymers were calculated using poly(methyl methacrylate)standards with *M*_w_ range from 17,000 to 250,000 g/mol and polydispersity lower than 1.14. The calculations were carried out using GPC LC Solutions software (Shimadzu, Kyoto, Japan).

The contribution of hydrophobic block was determined using chromatographic amino acid analysis after total hydrolysis of the samples. The hydrolyzate was analyzed by reversed-phase (RP) high-performance liquid chromatography (HPLC) using a Shimadzu system with UV-detection (Kyoto, Japan) equipped with 2 mm × 150 mm Luna C_18_column, packed with 5 μm particles. The isocratic elution mode was applied and 0.1% acetonitrile/НСООН in a ratio 5/95 wt % was used as eluent. The mobile phase flow rate was equal to 0.3 mL/min.

To study the influence of polymer composition, as well as different parameters used in the preparation step, the DLS method was used for particle size characterization. DLS measurements were performed on a SZ100 (Horiba JobinYvon, Kyoto, Japan) laser particle analyzer at a scattering angle of 90° at 25 °C. The range of concentrations of nanoparticles in Na-borate buffer solution, pH 8.7, was 0.5 and 0.25 mg/mL. The morphological peculiarities were investigated using transmission electronic microscopy (TEM) using a Jeol JEM-2100 (Tokyo, Japan) microscope operated at an acceleration voltage of 160 kV. Before analysis, a few drops of sample were placed onto a copper grid covered with carbon for 30 s. The dried grid was stained negatively with 2% (*w*/*v*) uranyl acetate solution for 30 s and used for measurements after 24 h.

### 2.3. Synthesis and Particles Preparation

NCAs of γ-Glu-(Bzl) and Phe were synthesized as described elsewhere [30]. Dioxane was used as a solvent, and acquired NCA was purified by recrystallization from ethyl acetate/n-hexane. Yields: NCA of γ-Glu-(Bzl)—75%, NCA of Phe—70%. Structure and purity level of synthesized NCAs were confirmed by ^1^H NMR. ^1^H NMR: NCA of γ -Glu-(Bzl)—2.05–2.39 (m, 2 H), 2.63 (t, 2 H), 4.39 (t, 1 H), 5.17 (s, 2 H), 6.40 (br. s., 1 H), 7.39 (m, 5 H), NCA of Phe: 2.94–3.35 (m, 2H), 4.55 (m, 1H), 5.60 (s, 1H), 7.19–7.41 (m, 5H). The ring opening polymerization (ROP) of γ-Glu-(Bzl) NCA was carried out using HEXA as initiator at 4% of monomer in 1,4-dioxane. The polymerization was carried out for 24 h at 30 °C. Then the polymer was precipitated, washed with diethyl ether three times, and dried. 

Then, p-Glu-(Bzl) copolymerization with Phe-NCA was carried out in DMF for 48 h at 30 °C to obtain a series of P-γ-Glu(Bzl)-*b*-PPhe with various P-Glu/PPhe balance. The Bzl-protective group of P-γ-Glu(Bzl)-*b*-PPhe was removed by TFMSA/TFA mixture in a ratio 1/10 at 22 °C. The samples were totally hydrolyzed and the products of hydrolysis were investigated by RP-HPLC as described in Section 2.2. The hydrolysis of 1 mg of a sample was carried out in 2 mL of 6 M HCl with 0.0001% phenol in vacuum-sealed ampoule for four days. The solvent was evaporated several times with water to eliminate HCl and to reach finally the neutral pH value.

After deprotection, the product was dispersed in DMSO, put into a dialysis membrane bag MWCO 1000, and dialyzed against Na-borate buffer solution, pH 8.6, for one day. After two days of freeze drying, PGlu-*b*-PPhe was collected. Polymer nanoparticles were prepared by phase inversion during dialysis, followed by freeze drying and final dispersing for 2 h under sonication at necessary concentration (0.25–1.00 mg/mL) in the corresponding buffer (Na-phosphate or Na-borate buffer solutions, pH 7.4–10.5). 

### 2.4. Biodegradation study

To study the biodegradation process of PGlu-*b*-PPhe nanoparticles, the accumulation of free amino acids in a medium was controlled. 0.02 M Na-phosphate buffer, pH 7.4, containing hydrolase papain, were used as model physiological conditions. For that, 50 μg of enzyme was introduced into suspension to reach the volume of 1 mL. In parallel, 1 mg of nanoparticles in 100 μL of 0.02 M Na-phosphate buffer, pH 7.4, was added to 900 μL of human blood plasma. All reactions of nanoparticle degradation were carried out for three months at 37°C and monitored by offline cation exchange HPLC of the reaction products (Glu and Phe amino acids). For this purpose, the commercially available methacrylate-based ultra-short monolithic column, namely, CIMSO_3_ disk (3 mm × 12 mm i.d.) (BIA Separations, Ajdovscina, Slovenia) was applied. UV detection was performed at 210 nm. The data was acquired and processed with LS Solution software (Shimadzu, Kyoto, Japan). 0.02 M aqueous Klark-Labbs buffer, pH 2.0, (eluent A), 0.02 M Na-phosphate buffer, pH 7.0, (eluent B) and 0.0125 M Na-borate buffer, pH 10.0 (eluent C), were used as the mobile phases for HPLC of Glu and Phe. The separation was carried out using follow protocol: 0–0.5 min—eluent A, 0.5–7 min—eluent B, 7–10 min—eluent C at a flow rate of 0.5 mL/min.

### 2.5. Surface Modification

The modification of the surface of PGlu_62_-*b*-PPhe_82_ nanoparticles with the model protein, namely, ribonuclease A, was carried out after preliminary activation of carboxylic groups. The nanoparticles were prepared in 0.01 M Na-borate solution, pH 8.4, and then dialyzed using a 10 kDa dialysis membrane against 0.01 M MES buffer, pH 6.0. 4 mL of suspension with a concentration of 0.5 mg/mL was mixed with a two-fold excess of NHS and CDI required to activate 20% of Glu units. The activation was carried out at 4 °C for 30 min. Then, 2 mg of ribonuclease was added to the suspension of activated nanoparticles and left under stirring conditions for 30 min at 20 °C. The excess of the enzyme was removed via dialysis using MWCO 30,000 membrane. The amount of immobilized enzyme was evaluated using the Lowry method [31]. The activity of bound and free ribonuclease was determined using low molecular weight-specific substrate 2,3-cytidine cyclophosphate and methodology published elsewhere [32]. 

### 2.6. Encapsulation of Dyes

For encapsulation of bromophenol blue, 4.0 mL solutions of dye with concentrations from 0.05 to 2.50 mg/mL in 0.01 M Na-borate buffer, pH 9.5, were prepared, then added to 4.0 mg of block copolymer, mixed, and sonicated for 4 h. The free dye was separated from particles by gel-filtration using a Sephadex G-100 gel column of 0.8 (i.d.) × 30.0 mm^2^. The part of encapsulated dye was calculated as a difference between total and free dye amounts, the latter was determined using spectrophotometric data measured at 590 nm and corresponding calibration curve. 

Rhodamin loading was performed as follows: 0.5 mg of rhodamin 6g dissolved in 0.4 mL DMSO, 1.0 mg of block copolymer and 3.6 mL of 0.01 Na-borate buffer, pH 10.5, were mixed and sonicated for 6 h. The excess of dye and DMSO were removed via dialysis through a 30 kDa MWCO membrane until no fluorescence was observed in a filtered solution.

### 2.7. Cell Experiments 

The cells were routinely cultured at 37 °C in a humidified atmosphere containing air and 5% CO_2_. Cytotoxicity of colloid solutions of polymer nanoparticles with concentrations 0.05–0.5 mg/mL was evaluated with a standard MTT test as described elsewhere [33]. The incubation of nanoparticles with cells was performed for 48 h. The values measured at 540 nm were subtracted for background correction at 690 nm, and the data was plotted as a percent of control samples using Microsoft Excel software (Microsoft Corp., Redmond, WA, USA).

To monitor if the particles penetrate the cells, 200 µL of cell culture medium containing Caco-2 cells were seeded on glass chamber slides (LabTec-II with CC2 treatment) and cultured for 24 h. Then, the incubation medium was changed with fresh one containing rhodamine-loaded polymersomes and the mixtures were incubated for 4 h. After that, the cells were washed three times with warm PBS solution and observed using a fluorescence microscope (Olympus IX50, Olympus Corp., Tokyo, Japan) equipped with a SC30 Olympus camera to capture the images of cells (excitation filter: BP 530–550, barrier filter: BA590). The images were acquired at 20× optical zoom.

## 3. Results and Discussion

### 3.1. Synthesis of Poly(Amino Acid) Block-Copolymers 

**Figure 1:**
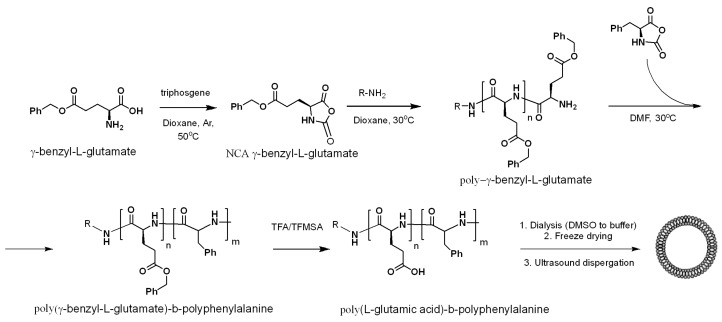
Scheme of synthesis of amphiphilic PGlu-*b*-PPhe copolymers for preparation of polymersomes.

The general scheme of polymer synthesis is presented in Figure 1. As a first step, the synthesis of several homopolymers of γ-Glu(Bzl) was carried out using the method of ring-opening polymerization of the corresponding NCA. 

The characteristics of synthesized P-γ-Glu(Bzl)s are collected in Table 1. As a second step, the P-γ-Glu(Bzl) was used as macroinitiator for copolymerization of the hydrophobic block of Phe-NCA. The formation of amphiphilic block copolymers was achieved after deprotection of the Bzl-protective group. Thus, four samples differing with the length of the hydrophilic and hydrophobic blocks were prepared (Table 2).

### 3.2. Characterization of Particles

To prepare the particles, “phase inversion” method, which was approved as the most suitable to obtain the nanoobjects with a polymersome structure [9], was applied. It is known that several factors influence the self-assembling behavior of amphiphilic polymers. Particularly, the length of hydrophobic/hydrophilic blocks, pH, and concentration are of great importance for the characteristics of the formed particles. 

**Figure 2:**
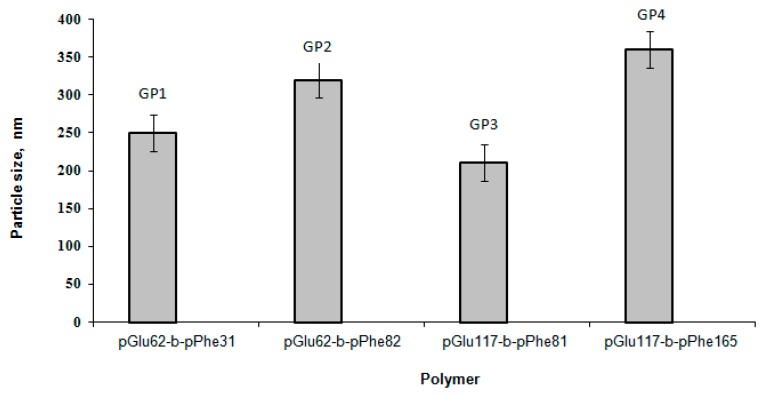
Effect of block length on particle size (particle concentration is 0.5 mg/mL, pH 8.4).

First of all, the effect of the block length on the particle hydrodynamic diameter was evaluated (Figure 2). The comparison of polymers containing the hydrophilic block of the same length, namely, GP1 with GP2 and GP3 with GP4, allowed the conclusion that the increase of the length of the hydrophobic block led to the formation of larger size particles. This result is in agreement with published data on self-assembly of other kinds of amphiphilic block-copolymers, for instance, poly(ethylene glycol)-*b*-poly(ε-caprolactone) [34] and poly(ethylene glycol)-*b*-polystyrene [35]. When the hydrophilic block of PGlu-*b*-PPhe was increased, but the hydrophobic block remained constant (samples GP2 and GP3) only a minor decrease in a particle hydrodynamic diameter was observed. This effect can be related to the higher repulsion of charged-like polymer chains with the growth of Glu-block length and, as a result, the self-assembly in a smaller sized particles. 

**Figure 3:**
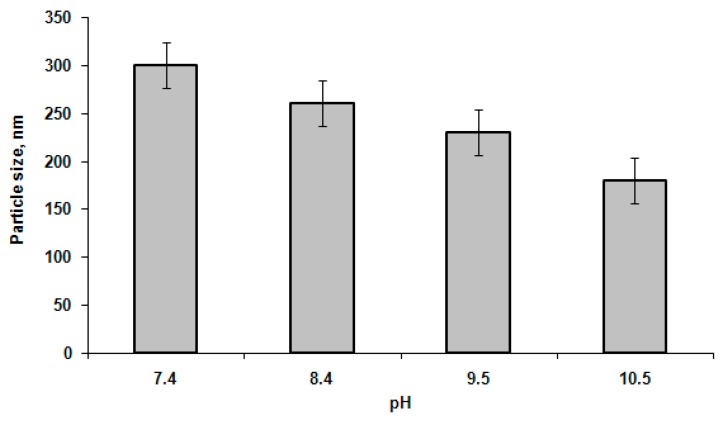
Dependence of particle size on pH (polymer sample GP1, concentration is 0.5 mg/mL).

The effect of pH on particle hydrodynamic diameter was examined using the series of 0.01 M buffer solutions with pH 7.4, 8.4, 9.5, and 10.5 and two polymer samples, namely GP1 and GP3. Since the tendency for both polymers was the same, the data for GP1 particles are presented as an example in Figure 3. It is obvious that the hydrodynamic diameter of nanoparticles slightly decreased in alkaline media. This can be attributed to the better PGlu solubility under alkaline conditions because of the higher ionization degree, which is favored to the formation of random coil conformation of polypeptides [36]. 

**Figure 4:**
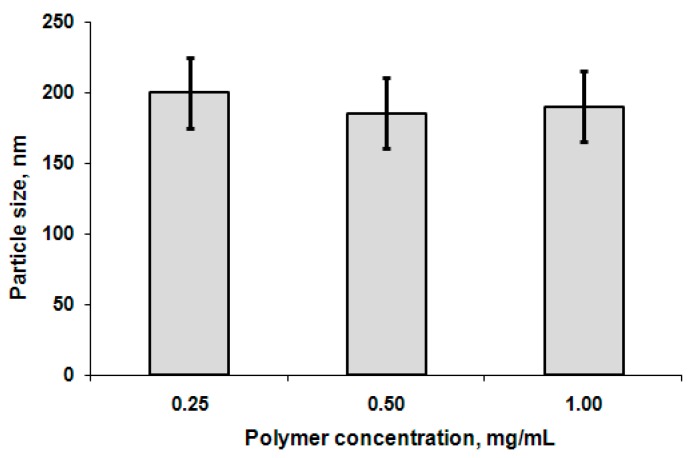
Dependence of particle size on polymer concentration (polymer sample GP3, pH 8.4).

**Figure 5:**
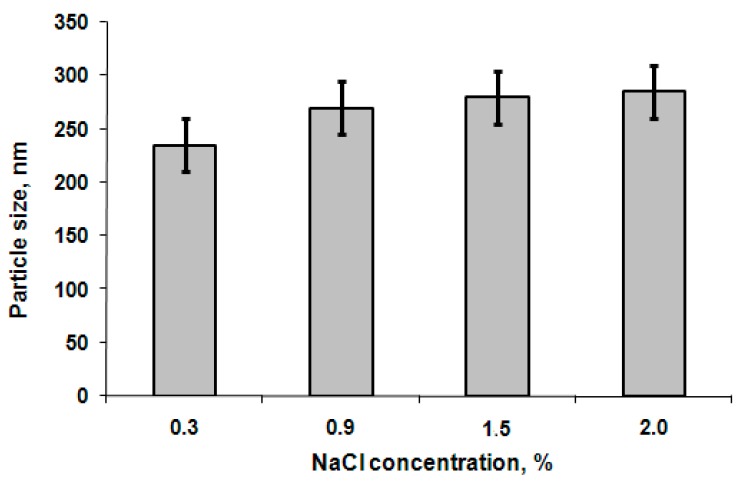
Effect of salt concentration on mean size of nanoparticles (polymer sample GP3, pH 7.4, particle concentration 0.5 mg/mL).

The effect of block copolymer concentration on particle hydrodynamic diameter was investigated within the following concentrations: 0.25, 0.50, and 1.00 mg/mL for all polymer samples. The defined values of nanoparticle hydrodynamic diameter did not show significant change with the growth of concentration. As an example, the DLS data for the samples prepared from GP3 copolymer are shown in Figure 4. Additionally, the influence of NaCl concentration was studied. No change in particle size was observed in the range of 0.3%–2.0% NaCl in 0.01 M Na-phosphate buffer (PBS), pH 7.4 (Figure 5). 

**Figure 6:**
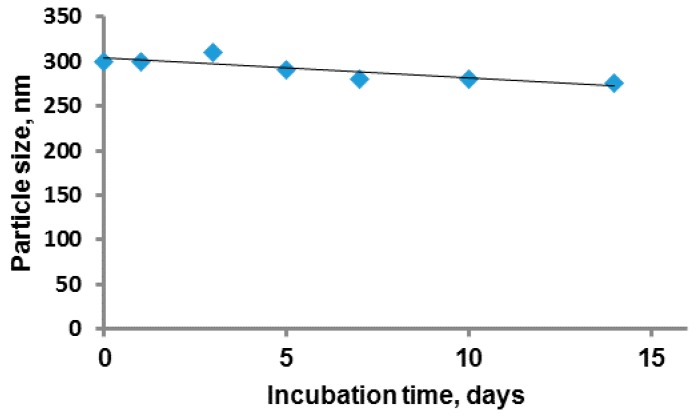
Storage stability of the GP3 colloid system at physiological conditions. Conditions: PBS buffer of pH 7.4; temperature 37 °C; concentration 0.5 mg/mL.

One of the most important characteristics of colloidal systems is ξ-potential, which reflects their stability regarding suspension aggregation. It is known that colloids are considered to be stable when the ξ-potential is lower than −30 and higher than +30 mV [37]. The values of ξ-potential for particles with hydrodynamic diameter up to 350 nm were around −70–50 mV at pH 7.4, which allowed assumption of their high stability. To examine the stability of PGlu-*b*-PPhe particles on aggregation, the experiments monitoring the particle size for two weeks at physiological conditions (PBS buffer, pH 7.4, 37 °C) were carried out (Figure 6). According to DLS data (Figure S1 of Supplementary Material), no other peaks, apart from a single one corresponding to initial-sized particles, were observed within the tested period. Thus, the developed nanoobjects can be characterized as a stable colloid system with no evident tendency to aggregation. 

**Figure 7:**
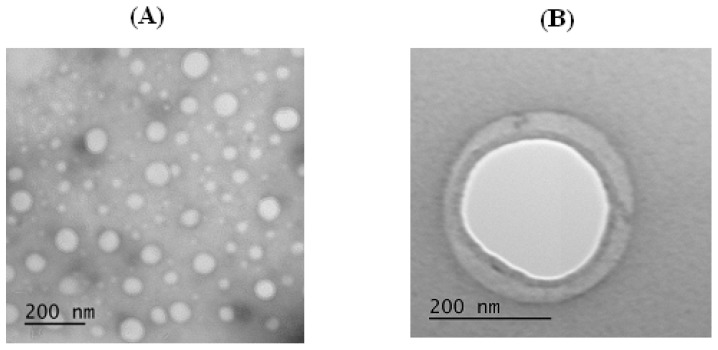
TEM images of polymer nanoparticles obtained (pH 8.4, concentration 0.5 mg/mL). (**A**) Polymersomes (GP2); and (**B**) single polymersome (GP3).

It is known that amphiphilic block copolymers can self-assemble into a wide range of morphologies [6]; for example, cylindrical, spherical micelles, or polymeric vesicles. The formed particles can be definitely identified as polymer vesicles, or polymersomes (Figure 7) possessing a polymer membrane (double dark circle) and aqueous core (light interior). Considering the hydrophobic block presented by polyphenylalanine, the observed structure can be formed due to π–π interactions between aromatic fragments of hydrophobic chains. Despite the copolymers applied to the preparation of particles differing with the length of the macromolecules and the length of hydrophobic block, the morphology for all samples prepared in the pH range 7.4–10.5 and concentration up to 1.0 mg/mL remained the same. 

### 3.3. Biodegradation 

The degradation of poly(amino acids) in biological environments occurs only when catalyzed by enzymes. The rate of degradation depends on the specificity of involved enzymes. The complication of enzymatic degradation is connected with the time-scale of the process. Even in the case of rapidly-degraded polymers in the presence of highly-active enzymes, the degradation rate can be affected by the decay of enzymatic activity. 

It is known that common extracellular proteinases of mammals, such as chymotrypsin A and elastase, both serine proteinases which are active towards the bonds formed with hydrophobic and neutral amino acids, are at least two orders of magnitude less active towards the charged poly(α-amino acids) comparatively to lyposomal endopeptidase cathepsin B (thiol proteinase with broad specificity) [38]. Papain, a thiol proteinase of plant origin, is known as the analog of cathepsin B regarding its activity towards the bonds formed between different α-amino acids.

**Figure 8:**
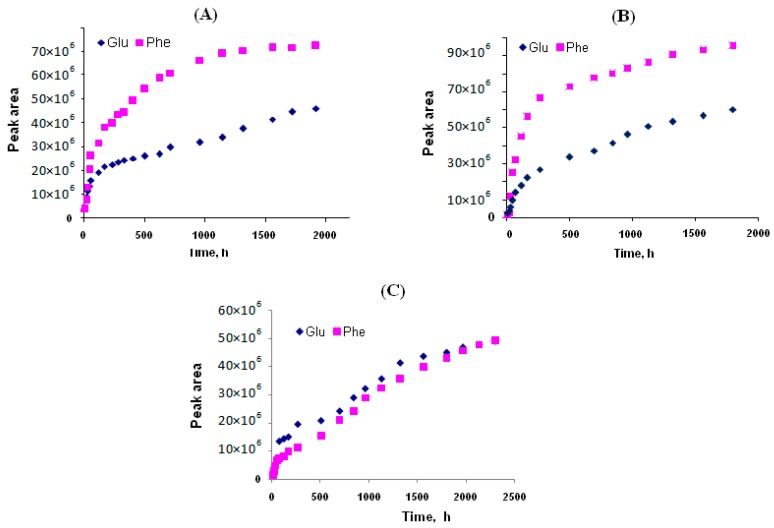
Accumulation of free amino acids during the biodegradation process of PGlu-*b*-PPhe polymersomes at physiological conditions (pH 7.4, 37 °C). (**A**) degradation of GP1 using papain; (**B**) degradation GP3 using papain; and (**C**) *in vitro* degradation of GP3 using blood serum plasma.

In our work, the biodegradation of prepared particles was studied using a model enzyme system and human blood plasma under physiological conditions. Papain was chosen as a model proteolytic enzyme. The degradation process was studied as a function of free amino acids accumulation in the reaction mixture. As it is seen from Figure 8, the degradation of both PGlu and PPhe catalyzed with papain occurred simultaneously. As expected, in the case of copolymer with shorter blocks, the degradation curve plateau was achieved faster. In this case, the degradation of polymer particles was practically finished after 45 days. At the same time, the degradation of polypeptide with longer chain blocks reached the plateau after approx. 60 days of degradation. 

*In vitro* degradation of the particles in blood plasma was less effective, and even after two months the total degradation was not achieved. It can be related to the lower activity and, probably, partial plasma enzyme inactivation during so long an incubation process. Another difference of the degradation in physiological medium was the most effective degradation of the PGlu block compared to the PPhe one, which can be related to the differences in the biocatalyst nature present in the systems. In any case, it can be concluded that the degradation of poly(amino acid)-based polymersomes at pH 7.4 and 37 °C is not quick, and takes several weeks. 

### 3.4. Surface Modification

The surface functional groups of the discussed nanoparticles open the wide possibility for their modification. It is known that proteins and peptides are often used as vector ligands for the targeted drug delivery [39]. In this work the labile enzyme ribonuclease A was used for functionalization of GP2 nanoparticles. The immobilization capacity was equal to 0.7 mg/mg of particles. The evaluation of the enzyme activity was performed using thecytidine-2’,3’-cyclophosphate as a specific low molecular weight substrate. The comparison of activity of free and bound forms of biocatalyst allowed the conclusion that the applied method of biofunctionalization did not contribute to the enzyme inactivation (Table 3). Additionally, the results on modification of GP3 with α-chymotrypsin can be found in the Supplementary data (Table S1). The activity of this enzyme was studied as described elsewhere [40]. 

### 3.5. Encapsulation of Model Compounds and Cell Experiments

Loading of model compounds, namely, bromophenol blue and rhodamine 6g, into polymersomes was carried out to prove the applicability of these kinds of particles as potential nanocontainers for drug delivery. 

**Figure 9:**
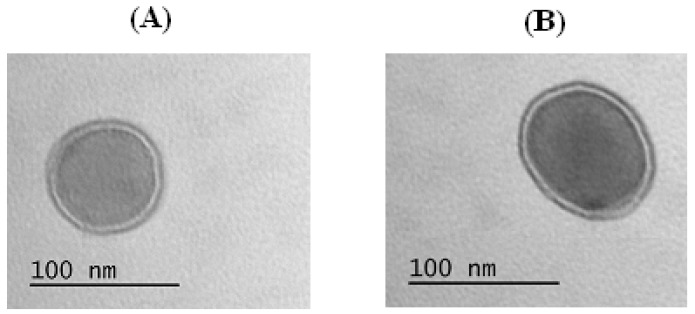
TEM images of GP2 polymersomes loaded with model dye (bromophenol blue): (**A**) encapsulation efficiency 16%; and (**B**) encapsulation efficiency 21%.

The encapsulation efficiency of bromophenol blue was in the range 8%–21% and depended on the process conditions. The encapsulation efficiency remained the same (about 20%) if the initial dye concentration did not exceed 1.0 mg/mL. In contrast, this value (%) linearly decreased to 8% if the initial concentration was increased up to 2.0–2.5 mg/mL (Figure S2 of Supplementary Material). The amount of encapsulated dye (μg) reached the plateau, when the initial concentration of the model compound was above 1.0 mg/mL. The maximum amount of loaded dye was 0.8 mg/mg of particles (Figure S3 of Supplementary Material). Qualitatively, the approval of dye encapsulation into polymersomes was shown by TEM images (Figure 9). The native polymersomes have a light core, while the loaded nanovesicles are dark inside due to bromophenol increasing their inner electronic density.

**Figure 10:**
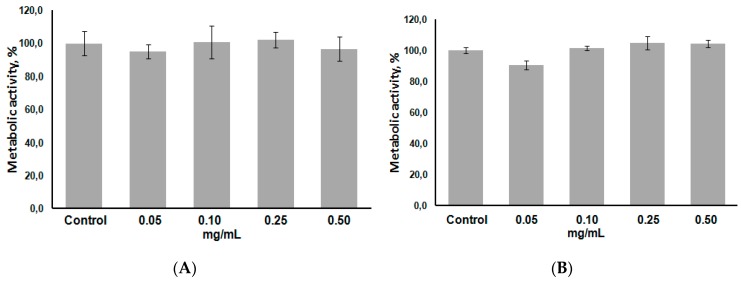
Viability of HEK (**A**) and Caco-2 (**B**) cells cultured in medium containing different concentrations of GP3 polymersomes.

To test the biocompatibility and cytotoxicity of PGlu-*b*-PPhe polymersomes, MTT assay using HEK and Сaco-2 cell lines was performed. The suspensions on PGlu_62_-*b*-PPhe_82_ (GP2) and PGlu_117_-*b*-PPhe_81_ (GP3) at four different concentrations, ranging from 0.05 to 0.50 mg/mL, were incubated with cells within 48 h. According to the results illustrated in Figure 10 for particles prepared from longer chain polymer no cytotoxicity was observed during the experimental time at all tested concentrations. The results obtained for the particles prepared from polymer with shorter PGlu chain absolutely coincided with those obtained for PGlu_117_-*b*-PPhe_81_-based polymersomes.

**Figure 11:**
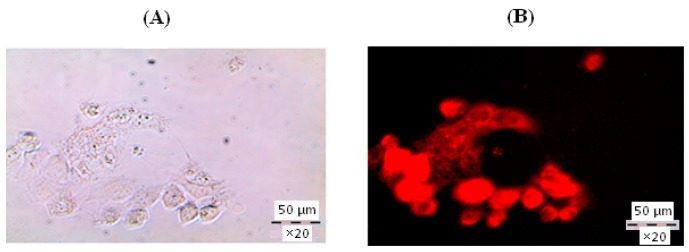
Bright-field (**A**) and fluorescent (**B**) images of Caco-2 cell containing GP3 particles stained with rhodamine 6g.

To investigate the penetration of particles obtained into the cells, the fluorescent dye rhodamine 6g was encapsulated into PGlu-*b*-PPhe polymersomes and incubated with the Caco-2 cells within four hours. The visualization of cells was done by fluorescent microscopy. The coloration of inner space of the cells with encapsulated dye proved the cellular uptake of developed polymersomes (Figure 11). 

## 4. Conclusions

The preparation of polymersomes from amphiphilic block-copolymer of glutamic acid and phenylalanine was studied. The structures obtained possessed the self-assembled membrane, the surface capable for biofunctionalization, and the core appropriate for encapsulation of hydrophilic compounds. The diameter of prepared polymersomes depended on pH of the solution used for self-assembly, as well as on the length of the hydrophobic block. Particularly, the mean size of polymersomes was managed to vary from 60 to 350 nm depending on the conditions of preparation and on the polymer chain length. Such important features of developed polymersomes as the absence of cytotoxicity and biodegradability, easy possibilities to functionalize the polymersome surface with protein without its inactivation, the capability to encapsulate the hydrophilic compound inside the nanoparticles and to deliver them into the cells, make this kind of polymer nanoconstruction quite attractive for further development of specifically-targeted drug delivery formulations. 

## Figures and Tables

**Table 1:** Molecular mass characteristics of P-γ-Glu(Bzl).

Sample	*M*_n_	*M*_w_	*M*_w_*/M*_n_	*n*
BG1	13,800	16,600	1.2	62
BG2	25,800	36,100	1.4	117

**Table 2:** Molecular mass characteristics of synthesized amphiphilic PGlu-*b*-PPhe copolymers.

Sample	*M*_n_ (PGlu)	*n*	*M*_n_ (PPhe)	*m*	Hydrophilic block content, %
GP1	8,000	62	4,600	31	64
GP2	8,000	62	12,100	82	40
GP3	15,100	117	11,900	81	56
GP4	15,100	117	24,200	165	38

**Table 3:** Kinetic parameters of cytidine-2’,3’-cyclophosphate hydrolysis catalyzed by ribonuclease.

Biocatalyst form	Activity, µmol·min^−1^·mg^−1^	*K*_M_, mM
Free ribonuclease A	2.4	27
Immobilized ribonuclease A	2.2	18

## Supplementary Materials

Supplementary Materials can be found at www.mdpi.com/2073-4360/8/6/212/s1.

## Abbreviations

The following abbreviations are used in this manuscript:

GPC: Gel permeation chromatography
TEM: Transmission electron microscopy
NCA: *N*-carboxyanhydrides
ROP: Ring-opening polymerization
HEXA: Hexylamine
DMF: Dimethylformamide
DMSO: Dimethyl sulfoxide
TFMSA: Trifluoromethane sulfonic acid
TFA: Trifluoroacetic acid
NHS: *N*-hydroxysuccinimide
CDI: 1-Ethyl-3-(3-dimethylaminopropyl)carbodiimide
MTT: 3-(4,5-dimethylthiazol-2-yl)-2,5-diphenyltetrazolium bromide
NMR: Nuclear magnetic resonance
DLS: Dynamic light scattering
HPLC: High performance liquid chromatography
HEK cells: Human embryonic kidney cells
Caco2 cells: Human epithelial colorectal adenocarcinoma cells
