# Supplementary material for: Preparation, Characterization, and Biological Evaluation of Poly(Glutamic Acid)-b-Polyphenylalanine Polymersomes
Source: Polymers (Basel). 2016 Jun 2;8(6):212. doi: 10.3390/polym8060212 (PMC6432269; doi:10.3390/polym8060212)
Supplement: Supplementary file 1 [file polymers-08-00212-s001.pdf]

Supplementary Materials: Preparation, Characterization and Biological Evaluation of Poly(Glutamic Acid)-*b*-Polyphenylalanine Polymersomes

Evgenia Vlakh, Anastasiia Ananyan, Natalia Zashikhina, Anastasiia Hubina, Aleksander Pogodaev, Mariia Volokitina, Vladimir Sharoyko and Tatiana Tennikova

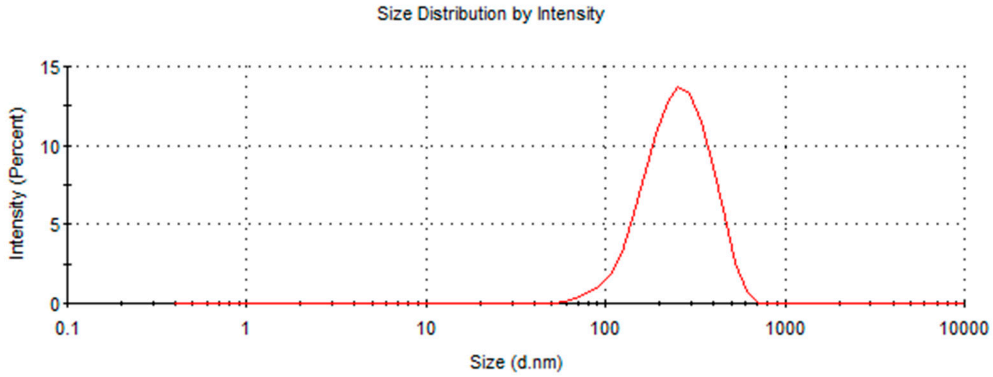

1 day

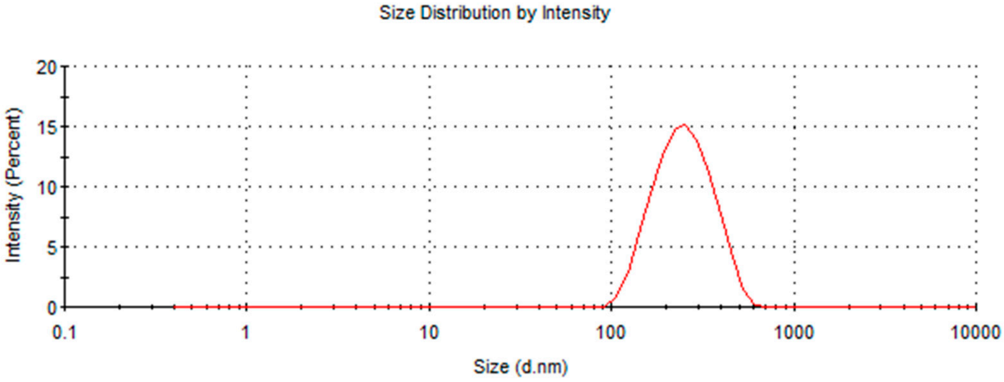

2 days

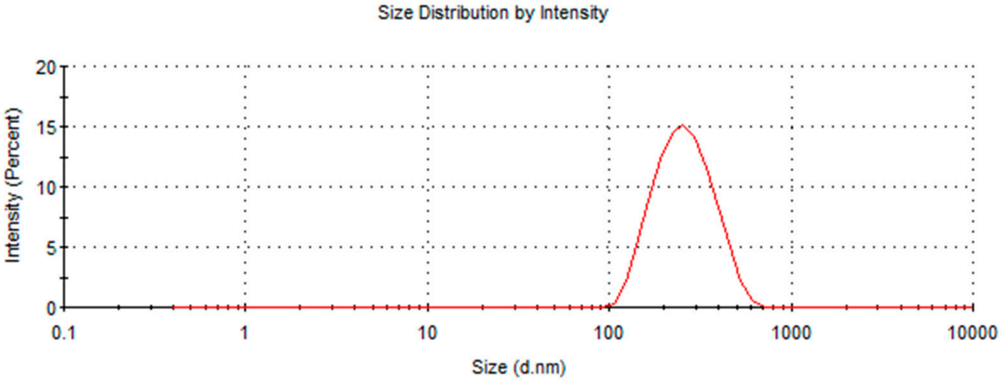

3 days

Figure S1. *Cont.*

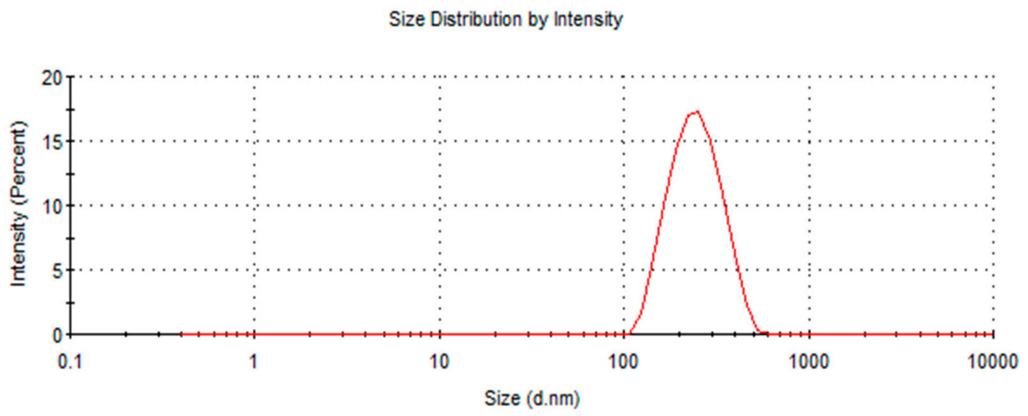

14 days

Figure S1. The DLS curves illustrating the particle hydrodynamic diameter distribution during the two weeks.

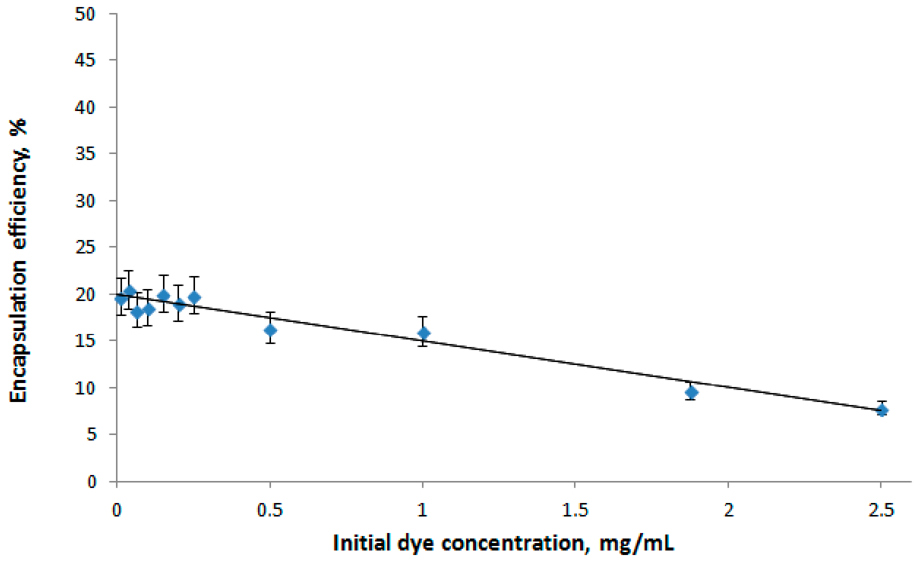

Figure S2. The dependence of encapsulation efficiency of bromophenol blue inside pGlu$_{62}$-*b*-pPhe$_{82}$ based particles on initial dye concentration (pH 9.5).

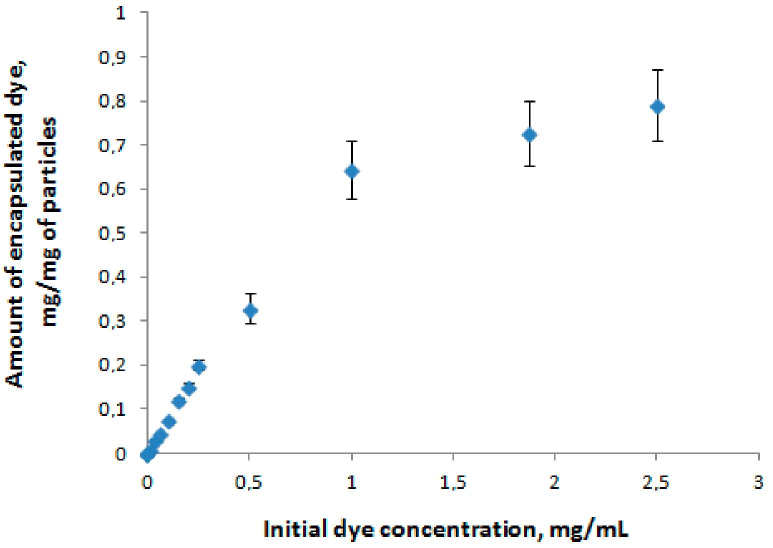

Figure S3. The dependence of bromophenol blue amount encapsulated inside pGlu$_{62}$-*b*-pPhe$_{82}$ based particles on initial dye concentration (pH 9.5).

Table S1. Kinetic parameters of benzoyl-L-tyrosine ethyl ester (BTEE) hydrolysis catalyzed by α-chymotrypsin (free and bound to the $pGlu_{117}$-*b*-$pPhe_{81}$ particles).

Biocatalyst Form	Activity, μmol·min^{-1}·mg^{-1}	K_M, mM
Free α-chymotrypsin	11.6	3.5
Immobilized α-chymotrypsin	19.6	9.5

The determination of enzyme activity was carried out as described in [40].

© 2016 by the authors; licensee MDPI, Basel, Switzerland. This article is an open access article distributed under the terms and conditions of the Creative Commons Attribution (CC-BY) license (http://creativecommons.org/licenses/by/4.0/).
